# Isogeometric analysis of wing structures using multipatch parametrization and penalty-based coupling method

**DOI:** 10.1038/s41598-026-42935-9

**Published:** 2026-03-06

**Authors:** Dawei Wang, Xian Cao, Yang Xue, Wei Wang, Xiaoxiao Du

**Affiliations:** 1 COMAC Beijing Aircraft Technology Research Institute, Beijing, 102211 China; 2 School of Mechanical Engineering and Automation, Beihang University, Beijing, 100191 China

**Keywords:** Geometric parametrization, Analysis-suitable, Isogeometric analysis, Wing structure, Reissner–Mindlin shell theory, Penalty-based coupling, Engineering, Mathematics and computing

## Abstract

Geometric parametrization and structural analysis play a pivotal role in aircraft structural design. Isogeometric analysis utilizes high-order, high-continuity spline basis functions for field variable discretization, offering superior solution accuracy compared to traditional finite element methods while streamlining data exchange between CAD and CAE systems. In this study, an analysis-suitable geometric parametrization is developed for classical aircraft wing structures, comprising two skins, twenty three ribs, and two spars. The wing geometry is represented using multiple NURBS patches. Within the wingbox formed by ribs and spars, all patch interfaces maintain conformity. Conversely, interfaces between wing skins and the wingbox are deliberately nonconforming to reduce modeling complexity while preserving the skin’s high continuity. Reissner–Mindlin shell theory is applied within the isogeometric framework to model structural behavior, and a penalty-based method is implemented to enforce continuity of the displacement field across the nonconforming skin-wingbox interface. A static bending analysis of the complete wing structure is performed, and the results are compared with those obtained from a conventional finite element analysis in ABAQUS for validation. The IGA results show excellent agreement with the FEA reference solution, achieving comparable accuracy with significantly fewer degrees of freedom.

## Introduction

Shape parametrization plays a pivotal role in structural design and aerodynamic shape optimization^1–3^. Classical structural shape optimization typically involves three interconnected models: the design model, which incorporates shape parametrization; the analysis model, which relies on mesh generation; and the optimization model, which establishes the relationship between the design and analysis models. Wing structural design represents a canonical application of shape parametrization, and numerous techniques have been developed for representing airfoil geometry, including Bézier curves, B-splines^4^, NURBS^5^, class function/shape function (CST) methods^6^, adaptive free-form deformation (FFD)^7^, and sensitivity-based geometric parametrization approaches^8^. These parametrization techniques are often integrated with mesh deformation algorithms, computational fluid dynamics (CFD), and optimization frameworks for aerodynamic design. In this study, we develop a NURBS-based shape parametrization method for geometric representation of both the airfoil and the complete wing structure, tailored for compatibility with isogeometric structural analysis.

Isogeometric analysis (IGA), introduced by Hughes et al.^9^, is an advanced numerical framework for computer-aided engineering that aims to unify geometric design and numerical analysis. By bypassing the traditional mesh generation process, IGA establishes a direct link between the design model and the analysis model, thereby significantly enhancing design efficiency. Unlike conventional finite element methods that employ Lagrange or other low-order shape functions, IGA leverages high-order, high-continuity spline basis functions, such as B-splines and NURBS, for field approximation. Owing to its ability to represent complex geometries exactly and its use of smooth basis functions, IGA has gained wide adoption in structural analysis and optimization^10–12^. In particular, IGA enables a direct coupling between design variables and simulation outputs in structural shape optimization. This facilitates the analytical derivation and efficient computation of sensitivities of objective functions with respect to design variables, thereby accelerating the convergence of the optimization process. Moreover, the similar mathematical foundations of NURBS approximations and BEM boundary descriptions have also led to extensive developments in isogeometric boundary element methods^5,13,14^.

The Kirchhoff–Love and Reissner–Mindlin shell theories are the two most widely used formulations in shell structure analysis^15^. The Kirchhoff–Love theory assumes that shell cross-sections remain straight and perpendicular to the mid-surface after deformation, neglecting transverse shear deformation. As a result, it is best suited for thin shell structures and requires at least $C^1$ continuity across element interfaces. A key advantage of this theory is its computational efficiency, as it involves only three translational displacement degrees of freedom (DOFs). In contrast, the Reissner–Mindlin shell theory relaxes the perpendicularity assumption and accounts for transverse shear deformation, making it applicable to both thin and thick shell structures. It requires only $C^0$ continuity across element boundaries but introduces additional computational cost due to the inclusion of three rotational DOFs, in addition to the three translational DOFs. In recent years, both theories have been effectively incorporated into isogeometric analysis frameworks for shell structures, leveraging their respective strengths^16–20^.

In engineering practice, complex shell structures are often constructed by using trimming and stitching operations during the design phase, and composed of multiple patches. During the design phase of shell structures, trimming and stitching operations can introduce geometric imperfections such as gaps or overlaps between patches, complicating the direct application of isogeometric analysis^21,22^. To mitigate these discontinuities, weak coupling methods, such as the penalty^11,23^, mortar^24,25^, and Nitsche’s method^26,27^, are widely adopted to enforce inter-patch continuity despite the presence of such defects. However, these approaches may occasionally lead to convergence or stability issues. Alternatively, unstructured parametrization approaches ( e.g., unstructured T-splines^28–30^, subdivision surfaces^31,32^, G-spline^33^, GNURBS^34^), have garnered significant attention in recent years due to their strong capability for representing complex geometries with extraordinary points.

In wing structure design, components including skins, ribs, and spars are conventionally modeled as separate entities in CAD systems. Based on the wing configuration, ribs and spars are positioned between the upper and lower skins, resulting in a non-manifold geometric configuration. While skins are generated through surface skinning techniques using airfoil profiles and wing configurations, ribs and spars are typically created via trimming operations relative to the skin surfaces, a process that presents challenges for isogeometric analysis. This paper proposes a hybrid conforming/non-conforming modeling strategy for analysis-suitable parametrization of wing structures. The approach enforces conforming parametrization within skin and wingbox while permitting controlled non-conforming interfaces at their junctions. An error-controlled NURBS parametrization is established for the airfoil profile, which provides a foundation for modeling of skin panels and wingbox components. The resulting wing structure representation intrinsically eliminates trimming features, a persistent source of numerical instabilities in isogeometric analysis. Structural analysis is performed within the isogeometric framework using Reissner–Mindlin shell theory, with displacement continuity at non-conforming interfaces enforced through an efficient penalty method. The methodology is rigorously validated through comprehensive convergence studies comparing IGA results for the RAE-2822 wing structure against finite element analysis results from commercial ABAQUS software. The developed integrated design and analysis framework is also extended to model and analyze six additional wing configurations featuring diverse airfoil profiles (structures using NACA-0012, NACA-6409, NACA-63015a, Davis B-24, AG-16, and A-18), confirming its robustness across different geometric configurations.

## Methods

**Fig. 1:**
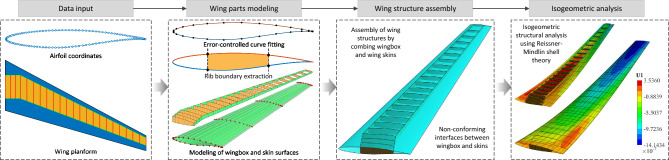
Overview of analysis-suitable modeling and structural analysis of aircraft wings.

This section details the methodology for analysis-suitable modeling and weakly coupled isogeometric analysis of wing structures. As illustrated in Fig. 1, the framework begins with predefined airfoil coordinates and a wing planform. An error-controlled fitting method is first employed to construct NURBS curves from the airfoil data. These curves are then used to generate the outer skin surfaces via a skinning technique. Subsequently, the internal ribs and spars are constructed based on the planform and the generated skin surfaces. The complete wing model is assembled from these skins, ribs, and spars. Finally, the structural performance is evaluated using an isogeometric Reissner-Mindlin shell analysis.

### Airfoil shape parametrization

**Fig. 2:**
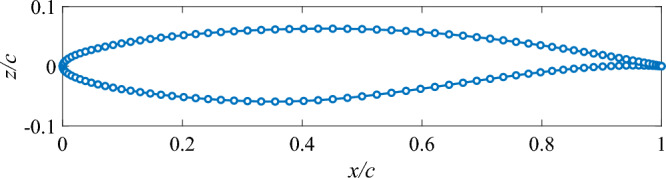
RAE 2822 airfoil and its 130 data points reproduced from^35^.

The RAE 2822 airfoil is first selected as the baseline geometry for the wing structure. Figure 2 illustrates the airfoil profile along with 130 discrete data points reproduced from^35^. These data points are partitioned into upper and lower surfaces, starting from the leading edge and extending to the trailing edge, to facilitate parametrization. Various airfoil shape parametrization techniques have been proposed in the literature^36,37^. In this study, a NURBS-based approach is adopted. Specifically, two NURBS curves are employed to fit the upper and lower surface data points, respectively. To simplify airfoil parametrization, all control point weights are set to 1. In this case, NURBS are mathematically equivalent to B-splines.

**Fig. 3:**
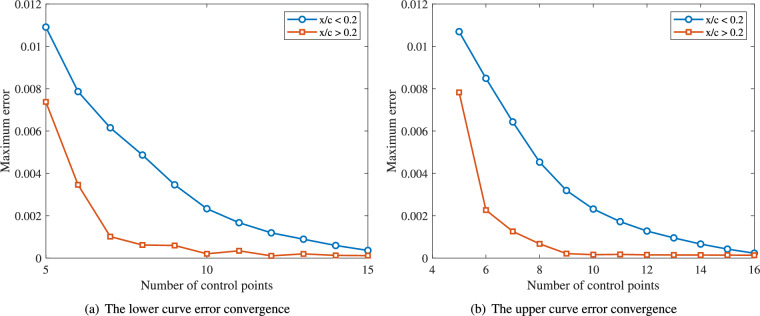
Relationship between the number of control points and maximum fitting error during the parametrization process.

A NURBS curve with $n+1$ control points ${{\boldsymbol{b}}}_i (i=0,1,\ldots ,n)$ could be defined as

1 $$\begin{aligned} {{\boldsymbol{C}}}(\xi ) = \sum _{i=0}^n R_{i,p}(\xi ) {{\boldsymbol{b}}}_i, \quad R_{i,p}(\xi ) = \dfrac{w_i N_{i,p}(\xi )}{ \sum _{j=0}^n w_j N_{j,p}(\xi ) }, \quad \xi \in [0,1], \end{aligned}$$

in which *p* is the degree, $R_{i,p}$ and $w_i$ are the NURBS basis function and weight corresponding to the *i*-th control point ${{\boldsymbol{b}}}_i$. $N_{i,p}(\xi )$ is the *i*-th B-spline function defined recursively by

2 $$\begin{aligned} N_{i,p}(\xi )=\dfrac{\xi -\xi _i}{\xi _{i+p}-\xi _i}N_{i,p-1}(\xi )+\dfrac{\xi _{i+p+1}-\xi }{\xi _{i+p+1}-\xi _{i+1}}N_{i+1,p-1}(\xi ), \end{aligned}$$

for $p = 1,2,\ldots$, and for $p=0$

3 $$\begin{aligned} N_{i,p}(\xi )= {\left\{ \begin{array}{ll} 1 & if \ \ \xi _i\le \xi \le \xi _{i+1}, \\ 0 & otherwise. \end{array}\right. } \end{aligned}$$

Given a set of $m+1$ data points ${{\boldsymbol{Q}}}_{k} (k=0,1,\ldots , m)$, the control points of the NURBS curve can be determined by minimizing the least-squares fitting error, defined as

4 $$\begin{aligned} min: \ f = \sum _{i=0}^{m} \left| \left| {{\boldsymbol{Q}}}_{k} - {{\boldsymbol{C}}}(\bar{\xi }_k) \right| \right| ^2 = \sum _{k=0}^{m} \left| \left| {{\boldsymbol{Q}}}_{k} - \sum _{i=0}^{n} R_{i,p}(\bar{\xi }_k) {{\boldsymbol{b}}}_i \right| \right| ^2, \end{aligned}$$

where $\bar{\xi }_k \in [0,1]$ is the parameter related to the *k*-th data point ${{\boldsymbol{Q}}}_{k}$ and the chord length parametrization scheme is utilized in this paper. Based on the parametrization of the data points, the classical knot placement technique^38^ is employed to generate the knot vector. By differentiating the fitting error with respect to each control point ${{\boldsymbol{b}}}_i$, the following system of equations is obtained and can be solved to determine the optimal control points

5 $$\begin{aligned} {{\boldsymbol{N}}}^T {{\boldsymbol{N}}} {{\boldsymbol{b}}} = {{\boldsymbol{N}}}^T {{\boldsymbol{Q}}} \quad \rightarrow \quad {{\boldsymbol{b}}} = \left( {{\boldsymbol{N}}}^T {{\boldsymbol{N}}} \right) ^{-1} {{\boldsymbol{N}}}^T {{\boldsymbol{Q}}}, \end{aligned}$$

in which ${{\boldsymbol{b}}}$ is the vector of control points, ${{\boldsymbol{Q}}}$ is the vector of data points, ***N*** is the basis function matrix. Two constraints are imposed and incorporated into the system of equations: (1) ${{\boldsymbol{b}}}_0 = {{\boldsymbol{Q}}}_0, {{\boldsymbol{b}}}_n = {{\boldsymbol{Q}}}_m$; (2) ${{\boldsymbol{b}}}_1^x = {{\boldsymbol{b}}}_0^x$. The first constraint ensures that the two end control points coincide with the first and last data points, thereby enforcing curve interpolation at the boundaries. The second constraint enforces verticality of the first control polygon edge with respect to the *x*-axis, by requiring that the *x*-coordinates of the first and second control points be equal.

To evaluate an acceptable solutions, a typical wind-tunnel tolerance $\tau$ is selected as the geometric error and is defined as^6,36^

6 $$\begin{aligned} \tau = {\left\{ \begin{array}{ll} 4 \times 10^{-4}, & if \ x/c < 0.2, \\ 8 \times 10^{-4}, & if \ x/c \ge 0.2. \end{array}\right. } \end{aligned}$$

The error $\tau$ is calculated as the difference in the *z*-coordinate between the target curve and the data points. In this study, the error is evaluated by projecting the data points onto the NURBS curve, as expressed by

7 $$\begin{aligned} \tau = \min || {{\boldsymbol{Q}}}_k - {{\boldsymbol{C}}} (\xi ) ||. \end{aligned}$$

**Fig. 4:**
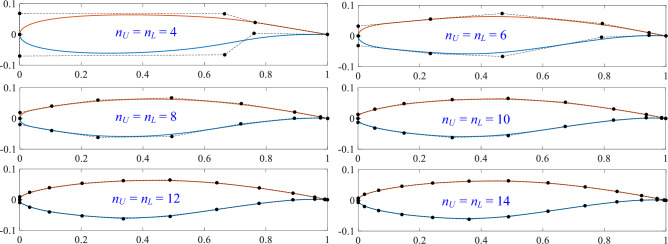
Convergence sequence of the airfoil fitting process with symmetric control point distribution $n_U = n_L =4,6,8,10,12,14$.

A polynomial degree of $p = 3$ is utilized as it represents the minimum requirement to maintain $C^2$ continuity (continuous curvature and torsion) across free-form curves. This level of continuity is essential for ensuring the aerodynamic smoothness of airfoil profiles. For applications requiring higher degrees, they can be precisely generated through the degree elevation algorithm without altering the geometric shape. The number of control points begins at 5 and increments by 1 until the fitting error tolerance $\tau$ is satisfied. Figure 3 depicts the convergence behavior of the maximum fitting error versus control point count for both lower and upper curves. Maximum fitting errors are analyzed separately for data points in the regions $x/c < 0.2$ and $x/c \ge 0.2$. In all cases, fitting errors decrease gradually as control points increase. The error tolerance $\tau$ is achieved at 15 control points for both the lower and upper curves. Figure 4 presents the progressive fitting results for the airfoil using control point counts $n_U = n_L =4,6,8,10,12,14$ for both upper and lower curves.

**Fig. 5:**
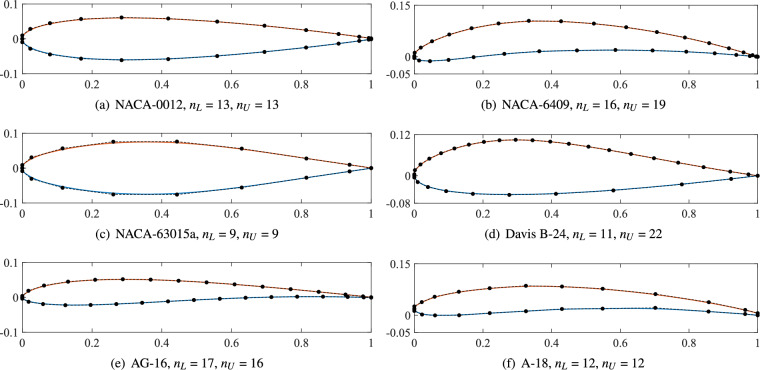
NURBS parametrization results for six airfoils (NACA-0012, NACA-6409, NACA-63015a, Davis B-24, AG-16, and A-18) within specified error tolerance.

**Fig. 6:**
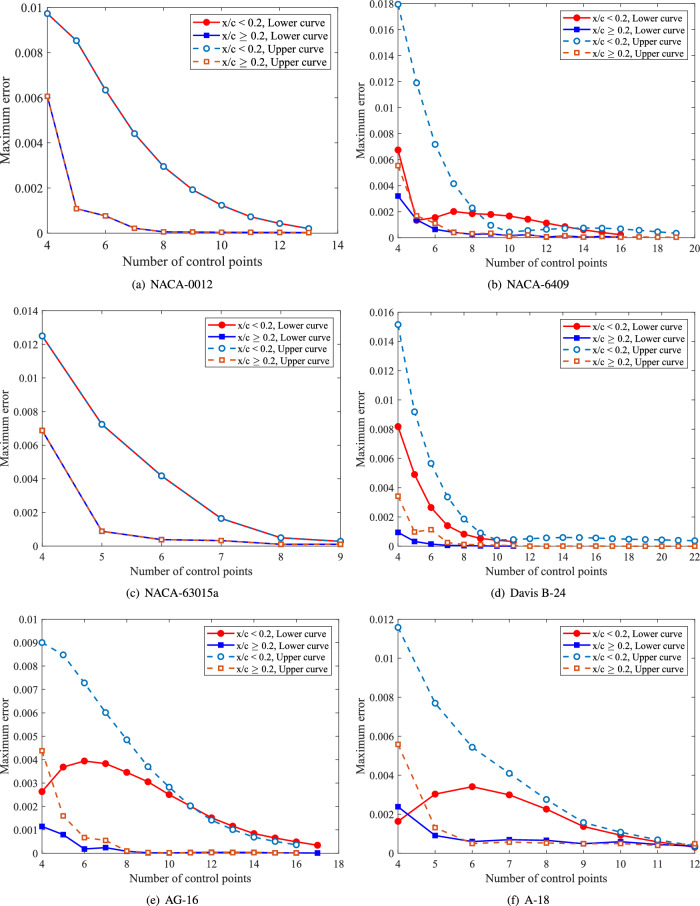
Convergence analysis of NURBS curve fitting for six airfoils (NACA-0012, NACA-6409, NACA-63015a, Davis B-24, AG-16, and A-18), showing maximum fitting error versus number of control points minus one.

In addition to the RAE-2822 airfoil, the proposed method is extended to six additional airfoil parametrizations, including the NACA-0012, NACA-6409, NACA-63015a, Davis B-24, AG-16, and A-18 airfoils. The data of these six airfoils is reproduced from^39^. Figure 5 illustrates the resulting airfoils represented by NURBS curves. The number of control points varies across airfoils. Symmetric airfoils (NACA-0012 and NACA-63015a) yield fitting curves that preserve geometric symmetry. The remaining four asymmetric airfoils exhibit distinct control point counts between upper and lower surfaces. Notably, the Davis B-24 airfoil requires 12 and 23 control points for its lower and upper curves, respectively, demonstrating a significant disparity. Figure 6 presents the convergence analysis of the fitting process of the six airfoils.

### Geometric parametrization of skin and wingbox

**Fig. 7:**
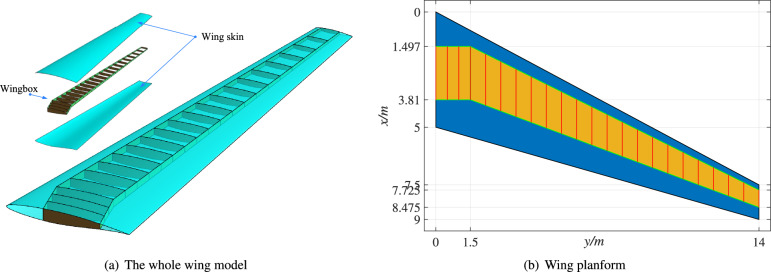
The RAE wing geometry (including a wing skin and wingbox) and its planform.

The wing model studied first in this paper is a benchmark widely used in aerostructural optimization, commonly referred to as the RAE benchmark wing^40^ or the MACH tutorial wing^41,42^. Herein termed the RAE wing (Fig. 7a), the model comprises wing skin (divided into upper and lower surfaces) and an internal wingbox structure. The wingbox consists of two spars and 23 ribs. The wing planform is designed to follow the Boeing 717 layout, as shown in Fig. 7b. All ribs are parallel to the *xz*-plane. The inboard four ribs are evenly distributed from *y* = 0 m to *y* = 1.5 m, while the outboard twenty ribs span evenly from *y* = 1.5 m to *y* = 14 m.

**Fig. 8:**
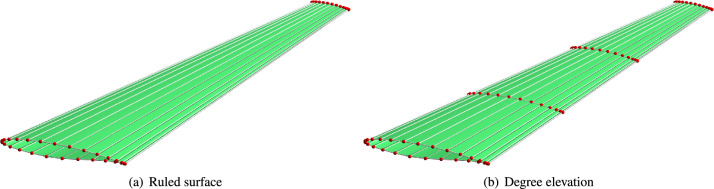
The wing skin surfaces are (**a**) linear and (**b**) cubic along the span direction.

Once the NURBS parametrization of the normalized airfoil is obtained (as described in the previous section), it is scaled to define the wing root and tip sections. The wing skin is then constructed by generating two ruled surfaces between these sections (Fig. 8a). While these two surfaces are linearly interpolated along the span direction, they are elevated to cubic interpolation as illustrated in Fig. 8b.

**Fig. 9:**
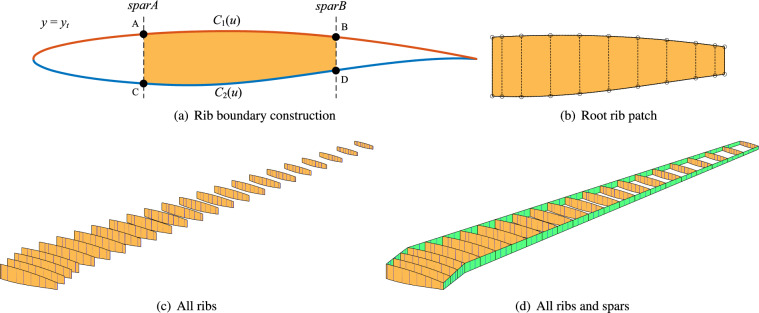
Modelling of wing ribs and spars. (**a**) Exact extraction of rib boundaries from the wing section curves, (**b**) construction of wing ribs using ruled surface scheme, (**c**) Generation of all ribs and (**d**) spars.

There are 23 ribs, each oriented parallel to the root section. The middle surface of each rib is a scaled version of the initial airfoil and can be generated by uniformly scaling the original airfoil curves. The two spars are oriented perpendicular to the *x*–*y* plane. For a rib located at the cross section $y = y_t$, assume the two spars are labeled *sparA* and *sparB*, intersecting the scaled airfoil at points *A*, *B*, *C* and *D*, as illustrated in Fig. 9a. These four intersection points are computed as the intersections of the lines $\overline{AC}$ and $\overline{BD}$ with the NURBS representation of the airfoil. This operation can be further simplified by considering intersections between these lines and the constituent Bézier curve segments. For the upper and lower airfoil surfaces, all Bézier curve segments are first extracted. Each segment is then checked for intersection with the specified lines. A bisection method is applied to refine the parametric domain iteratively until the intersection points are found to within a tolerance of $1\times 10^{-8}$. Once the intersection points and their parametric coordinates are determined, the curve segment between *sparA* and *sparB* is precisely extracted using the knot insertion algorithm. Let $C_1(u)$ and $C_2(u)$ denote the two extracted curve segments. Since these curves generally have different parametrizations, they are reparameterized to share a common knot vector. A ruled surface is then constructed between $C_1(u)$ and $C_2(u)$ to represent the rib at the section $y = y_t$, as shown in Fig. 9b. Figure 9a presents all 23 ribs, illustrating that each rib possesses a distinct parametrization.

For spar modeling, two NURBS patches are constructed to represent the spar domain between adjacent ribs. It should be noted that the interfaces between the spars and skins are generally curved. In this study, twenty equally spaced points are first generated along the line connecting the corresponding corner points of two adjacent ribs. These points are then projected onto the skin surfaces to obtain discrete interface points. Subsequently, a cubic NURBS curve with six control points and a uniform knot vector is fitted to these discrete points to accurately represent the interface. Figure 9b illustrates all ribs and spars. A total of 22 NURBS patches are used for both the leading and trailing spars. At this stage, the entire wing structure, comprising skins, ribs, and spars, is fully constructed.

**Fig. 10:**
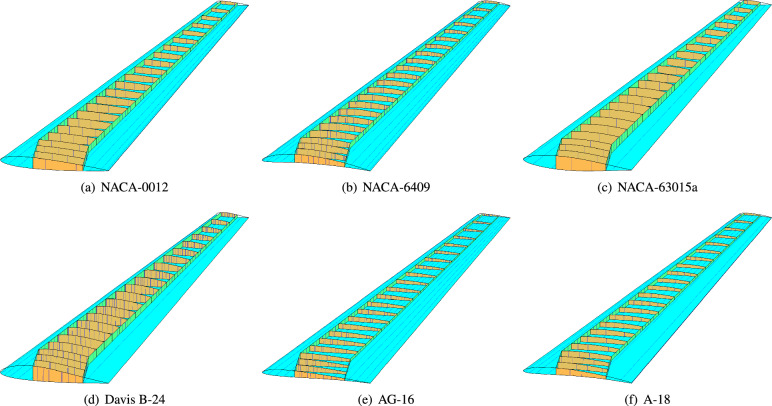
Parametric modeling of wing structures using RAE-2822 planform with varying airfoil sections: (**a**) NACA-0012, (**b**) NACA-6409, (**c**) NACA-63015a, (**d**) Davis B-24, (**e**) AG-16, (**f**) A-18.

The proposed parametrization method demonstrates extensibility beyond the RAE wing configuration. As illustrated in Fig. 10, we implement this approach using the RAE-2822 planform combined with the airfoil profiles from Fig. 5. The parametric curves corresponding to NURBS patch interior knots are highlighted in blue, while the upper skin surface is rendered in transparent cyan for enhanced visualization.

### Isogeometric Reissner–Mindlin shell formulations

This paper employs the isogeometric Reissner–Mindlin shell theory, which accounts for shear deformation effects. Under the Reissner–Mindlin assumptions, the director vector initially perpendicular to the mid-surface does not necessarily remain perpendicular after deformation. The position vector of any point within the shell structure can be expressed as:

8 $$\begin{aligned} {\boldsymbol{{x}}}(\xi ,\eta ,\zeta ) = \tilde{{\boldsymbol{{x}}}}(\xi ,\eta ) + \dfrac{\bar{t}}{2} \zeta {\boldsymbol{{n}}}(\xi ,\eta ), \quad \zeta \in [-1,1], \end{aligned}$$

in which ${\boldsymbol{{n}}}(\xi ,\eta )$ denotes the director vector of the middle surface $\tilde{{\boldsymbol{{x}}}}(\xi ,\eta )$, $\bar{t}$ is the shell thickness and $\zeta$ indicates the thickness parameter. Assuming the shell undergoes small displacements and small rotations, the displacement field within the shell can be expressed as

9 $$\begin{aligned} {\boldsymbol{{u}}}(\xi ,\eta ,\zeta ) = \tilde{{\boldsymbol{{u}}}}(\xi ,\eta ) + \dfrac{\bar{t}}{2} \zeta \left[ \tilde{\boldsymbol{\theta }}(\xi ,\eta ) \times {\boldsymbol{{n}}}(\xi ,\eta ) \right] , \end{aligned}$$

where $\tilde{{\boldsymbol{{u}}}}(\xi ,\eta )$ and $\tilde{\boldsymbol{\theta }}(\xi ,\eta )$ represent the translational and rotational displacements of the mid-surface, respectively. By employing NURBS basis functions to represent the mid-surface, both $\tilde{{\boldsymbol{{u}}}}(\xi ,\eta )$ and $\tilde{\boldsymbol{\theta }}(\xi ,\eta )$ can be approximated through interpolation of the translational and rotational degrees of freedom defined at the control points of the mid-surface, as follows

10 $$\begin{aligned} \tilde{{\boldsymbol{{u}}}}(\xi ,\eta ) = \sum _{I=1}^{n_{cp}} R_I(\xi ,\eta ) \tilde{{\boldsymbol{{u}}}}_I, \quad \tilde{\boldsymbol{\theta }}(\xi ,\eta ) = \sum _{I=1}^{n_{cp}} R_I(\xi ,\eta ) \tilde{\boldsymbol{\theta }}_I, \end{aligned}$$

Inserting Eq. (10) into Eq. (9), the displacement of the shell structures can be re-expressed as

11 $$\begin{aligned} {\boldsymbol{{u}}}(\xi ,\eta ,\zeta ) = \sum _{I=1}^{n_{cp}} R_I(\xi ,\eta ) \left\{ \tilde{{\boldsymbol{{u}}}}_I + \dfrac{\bar{t}}{2} \zeta \left[ \tilde{\boldsymbol{\theta }}_I \times {\boldsymbol{{n}}}(\xi ,\eta ) \right] \right\} . \end{aligned}$$

It is important to highlight that there are two approaches to compute the director vectors ${\boldsymbol{{n}}}(\xi ,\eta )$: the exact scheme and the interpolated scheme. The exact scheme yields an exact expression for the unit normal vector given by

12 $$\begin{aligned} {\boldsymbol{{n}}}(\xi ,\eta ) = \dfrac{ \tilde{{\boldsymbol{{x}}}}_{,\xi }(\xi ,\eta ) \times \tilde{{\boldsymbol{{x}}}}_{,\eta }(\xi ,\eta ) }{ || \tilde{{\boldsymbol{{x}}}}_{,\xi }(\xi ,\eta ) \times \tilde{{\boldsymbol{{x}}}}_{,\eta }(\xi ,\eta ) ||}, \end{aligned}$$

where $\tilde{{\boldsymbol{{x}}}}_{,\xi }(\xi ,\eta )$ and $\tilde{{\boldsymbol{{x}}}}_{,\eta }(\xi ,\eta )$ denote the partial derivatives of the mid-surface with respect to the two in-plane parametric directions. The interpolated scheme, on the other hand, utilizes the director vectors ${\boldsymbol{{n}}}_I$ defined at the control points to approximate ${\boldsymbol{{n}}}(\xi ,\eta )$, leading to the following expression for the displacement field

13 $$\begin{aligned} {\boldsymbol{{u}}}(\xi ,\eta ,\zeta ) = \sum _{I=1}^{n_{cp}} R_I(\xi ,\eta ) \left\{ \tilde{{\boldsymbol{{u}}}}_I + \dfrac{\bar{t}}{2} \zeta \left[ \tilde{\boldsymbol{\theta }}_I \times {\boldsymbol{{n}}}_I \right] \right\} . \end{aligned}$$

It should be noted that the control points of a mid-surface represented by NURBS do not generally lie on the surface itself. Consequently, the director vectors defined at the control points are often evaluated at corresponding collocation points on the mid-surface. Adam et al.^43^ recommend collocation at the Greville abscissae and reported a good compromise between accuracy and computational cost. In this paper, we employ the exact normal vector scheme due to its superior accuracy.

The small strain tensor is derived from the spatial derivatives of the displacement field as

14 $$\begin{aligned} \hat{\boldsymbol{\epsilon }}_g = \dfrac{1}{2}( {{\boldsymbol{u}}}_{,{{\boldsymbol{x}}}} + {{\boldsymbol{u}}}_{,{{\boldsymbol{x}}}}^T ), \end{aligned}$$

which is usually expressed in Voigt notation as $\boldsymbol{\epsilon }_g = [\epsilon _{11},\epsilon _{22},\epsilon _{33},2\epsilon _{12},2\epsilon _{13},2\epsilon _{23}]^T$ for procedure implementation. Inserting the displacement expression given in Eq. (13), the strain vector is rewritten as $\boldsymbol{\epsilon }_g = {{\boldsymbol{B}}} \bar{{{\boldsymbol{u}}}}$ with ${{\boldsymbol{B}}} = [{{\boldsymbol{B}}}_0,{{\boldsymbol{B}}}_1, \cdots , {{\boldsymbol{B}}}_n]$ and the component ${{\boldsymbol{B}}}_I$ is given by

15 $$\begin{aligned} {{\boldsymbol{B}}}_I = \begin{bmatrix} R_{I,x} & 0 & 0 & 0 & (\bar{R}_I n_3)_{,x} & -(\bar{R}_I n_2)_{,x} \\ 0 & R_{I,y} & 0 & -(\bar{R}_I n_3)_{,y} & 0 & (\bar{R}_I n_1)_{,y} \\ 0 & 0 & R_{I,z} & (\bar{R}_I n_2)_{,z} & -(\bar{R}_I n_1)_{,z} & 0 \\ R_{I,y} & R_{I,x} & 0 & -(\bar{R}_I n_3)_{,x} & (\bar{R}_I n_3)_{,y} & (\bar{R}_I n_1)_{,x}-(\bar{R}_I n_2)_{,y} \\ 0 & R_{I,z} & R_{I,y} & (\bar{R}_I n_2)_{,y}-(\bar{R}_I n_3)_{,z} & -(\bar{R}_I n_1)_{,y} & (\bar{R}_I n_1)_{,z} \\ R_{I,z} & 0 & R_{I,x} & (\bar{R}_I n_2)_{,x} & (\bar{R}_I n_3)_{,z}-(\bar{R}_I n_1)_{,x} & -(\bar{R}_I n_2)_{,z} \\ \end{bmatrix}, \end{aligned}$$

in which $\bar{R}_I = h \zeta R_I/2$. The stress vector is obtained by $\boldsymbol{\sigma }_g = {{\boldsymbol{D}}}_g \boldsymbol{\epsilon }_g$, where ${{\boldsymbol{D}}}_g$ is the global constitutive matrix and could be transformed from the local constitutive matrix ${{\boldsymbol{D}}}_l$ with ${{\boldsymbol{D}}}_g = {{\boldsymbol{T}}} {{\boldsymbol{D}}}_l {{\boldsymbol{T}}}^T$^44^. The transformation matrix ${{\boldsymbol{T}}}$ is computed from an orthonormal basis $\mathcal {V}_l = \{{{\boldsymbol{t}}}_1,{{\boldsymbol{t}}}_2,{{\boldsymbol{t}}}_3\}$ with

16 $$\begin{aligned} {{\boldsymbol{t}}}_1 = \dfrac{\tilde{{\boldsymbol{{x}}}}_{,\xi }(\xi ,\eta )}{||\tilde{{\boldsymbol{{x}}}}_{,\xi }(\xi ,\eta )||}, \quad {{\boldsymbol{t}}}_3 = {\boldsymbol{{n}}}(\xi ,\eta ), \quad {{\boldsymbol{t}}}_2 = {{\boldsymbol{t}}}_3 \times {{\boldsymbol{t}}}_1. \end{aligned}$$

Combining the above discretizations, the variation of the potential work of Reissner-Mindlin shell structures could be given by

17 $$\begin{aligned} \begin{aligned} \delta \mathcal {W}_{Tol}&= \delta \mathcal {W}_{Int} - \delta \mathcal {W}_{Ext} \\&= \delta {{\boldsymbol{u}}}^T \left[ \int _{V} \left( {{\boldsymbol{B}}}^T {{\boldsymbol{D}}}_g {{\boldsymbol{B}}} \right) dV \right] {{\boldsymbol{u}}} - \delta {{\boldsymbol{u}}}^T \left[ \int _{V} {{\boldsymbol{R}}}^T \bar{{{\boldsymbol{b}}}} dV + \int _{\Gamma } {{\boldsymbol{R}}}^T \bar{{{\boldsymbol{t}}}} d \Gamma \right] , \end{aligned} \end{aligned}$$

where $\bar{{{\boldsymbol{b}}}}$ is the body force, $\bar{{{\boldsymbol{t}}}}$ is the traction, *V* denotes the volume, $\Gamma$ indicates the boundary domain and ${{\boldsymbol{u}}}$ consists of translational and rotational displacements at all control points.

Finally, the bulk stiffness matrix ${{\boldsymbol{K}}}_b$ and the external force vector ${{\boldsymbol{F}}}$ are derived as

18 $$\begin{aligned} {{\boldsymbol{K}}}_b = \int _{V} {{\boldsymbol{B}}}^T {{\boldsymbol{D}}}_g {{\boldsymbol{B}}} d V, \quad {{\boldsymbol{F}}} = \int _{V} {{\boldsymbol{R}}}^T \bar{{{\boldsymbol{b}}}} d V + \int _{\Gamma } {{\boldsymbol{R}}}^T \bar{{{\boldsymbol{t}}}} d \Gamma , \end{aligned}$$

which can be further integrated in the parametric domain $\bar{V}$ and $\bar{\Gamma }$ using Jacobian transformations as

19 $$\begin{aligned} {{\boldsymbol{K}}}_b = \int _{\bar{V}} {{\boldsymbol{B}}}^T {{\boldsymbol{D}}}_g {{\boldsymbol{B}}} |{{\boldsymbol{J}}}_V| d \bar{V}, \quad {{\boldsymbol{F}}} = \int _{\bar{V}} {{\boldsymbol{R}}}^T \bar{{{\boldsymbol{b}}}} |{{\boldsymbol{J}}}_V| d \bar{V} + \int _{\bar{\Gamma }} {{\boldsymbol{R}}}^T \bar{{{\boldsymbol{t}}}} |{{\boldsymbol{J}}}_{\Gamma }| d \bar{\Gamma }. \end{aligned}$$

For the volume domain *V*, the Jacobian matrix ${{\boldsymbol{J}}}_V$ consists of the derivatives of the shell position with respect to parametric coordinates $(\xi , \eta , \zeta )$, namely, ${{\boldsymbol{J}}}_V = [{\boldsymbol{{x}}}_{,\xi }, {\boldsymbol{{x}}}_{,\eta }, {\boldsymbol{{x}}}_{,\zeta }]^T$ with

20 $$\begin{aligned} {\boldsymbol{{x}}}_{,\xi } = \tilde{{\boldsymbol{{x}}}}_{,\xi } + \dfrac{\bar{t}}{2} \zeta {\boldsymbol{{n}}}_{,\xi }, \quad {\boldsymbol{{x}}}_{,\eta } = \tilde{{\boldsymbol{{x}}}}_{,\eta } + \dfrac{\bar{t}}{2} \zeta {\boldsymbol{{n}}}_{,\eta }, \quad {\boldsymbol{{x}}}_{,\zeta } = \dfrac{\bar{t}}{2} {\boldsymbol{{n}}}. \end{aligned}$$

Then the determinant $|{{\boldsymbol{J}}}_V| = \left( {\boldsymbol{{x}}}_{,\xi } \times {\boldsymbol{{x}}}_{,\eta } \right) \cdot {\boldsymbol{{x}}}_{,\zeta }$. While for the surface domain $\Gamma$, the determinant $|{{\boldsymbol{J}}}_{\Gamma }| = |{\boldsymbol{{x}}}_{,\xi } \times {\boldsymbol{{x}}}_{,\eta }|$.

It is noted that the local constitutive matrix ${{\boldsymbol{D}}}_l$ can be explicitly given by

21 $$\begin{aligned} {{\boldsymbol{D}}}_l = \dfrac{E}{1-\nu ^2} \begin{bmatrix} 1 & \nu & 0 & 0 & 0 & 0 \\ \nu & 1 & 0 & 0 & 0 & 0 \\ 0 & 0 & 0 & 0 & 0 & 0 \\ 0 & 0 & 0 & (1-\nu )/2 & 0 & 0 \\ 0 & 0 & 0 & 0 & \kappa (1-\nu )/2 & 0 \\ 0 & 0 & 0 & 0 & 0 & \kappa (1-\nu )/2 \\ \end{bmatrix}, \end{aligned}$$

in which *E* and $\nu$ denote Young’s modulus and Poisson ratio, respectively, $\kappa$ is the shear correction factor, typically taken as 5/6. The third-row components of ${{\boldsymbol{D}}}_l$ are all zero to enforce the plane stress condition $\sigma _{33}^l = 0$, which may cause singularity in the global stiffness matrix. To prevent this singularity, a stabilization sub-matrix can be added to the rotational DOF positions in the global stiffness matrix^10,17^.

### Penalty-based multipatch coupling

In non-conforming multipatch modeling, coupling constraints between adjacent patches must be imposed to enforce both translational and rotational continuity. The variation of the coupling work resulting from these constraints can be decomposed into two components:

22 $$\begin{aligned} & \delta \mathcal {W}_t = \alpha _t \int _{\Gamma _C} \left( \tilde{{\boldsymbol{{u}}}} ^m - \tilde{{\boldsymbol{{u}}}} ^s \right) \left( \delta \tilde{{\boldsymbol{{u}}}}^m - \delta \tilde{{\boldsymbol{{u}}}}^s \right) d \Gamma , \end{aligned}$$

23 $$\begin{aligned} & \delta \mathcal {W}_r = \alpha _r \int _{\Gamma _C} \left( \tilde{\boldsymbol{\theta }}^m - \tilde{\boldsymbol{\theta }}^s \right) \left( \delta \tilde{\boldsymbol{\theta }}^m - \delta \tilde{\boldsymbol{\theta }}^s \right) d \Gamma , \end{aligned}$$

where $\alpha _t$ and $\alpha _r$ are the penalty parameters for coupling translational and rotational degrees of freedom, respectively. Pasch et al.^45^ proposed an a priori method for determining these penalty parameters based on the geometric dimensions and material properties of shell structures.

Considering the NURBS-based discretization, the coupling work gives rise to the following translational and rotational coupling stiffness matrices, ${{\boldsymbol{K}}}_c^t$ and ${{\boldsymbol{K}}}_c^r$, respectively

24 $$\begin{aligned} {{\boldsymbol{K}}}_c^t = \alpha _t \int _{\Gamma _C} \boldsymbol{\Phi } d \Gamma , \quad {{\boldsymbol{K}}}_c^r = \alpha _r \int _{\Gamma _C} \boldsymbol{\Phi } d \Gamma , \quad \boldsymbol{\Phi } = \begin{bmatrix} \boldsymbol{\Phi }_1^T \boldsymbol{\Phi }_1 & - \boldsymbol{\Phi }_1^T \boldsymbol{\Phi }_2 \\ - \boldsymbol{\Phi }_2^T \boldsymbol{\Phi }_1 & \boldsymbol{\Phi }_2^T \boldsymbol{\Phi }_2 \end{bmatrix}, \end{aligned}$$

in which $\boldsymbol{\Phi }_k (k=1,2)$ is the shape function matrix corresponding to the patch *k*, and is formed as

25 $$\begin{aligned} \boldsymbol{\Phi }_k = \begin{bmatrix} R_0 & 0 & 0 & R_1 & 0 & 0 & \cdots & R_{n_k}^k & 0 & 0 \\ 0 & R_0 & 0 & 0 & R_1 & 0 & \cdots & 0 & R_{n_k}^k & 0 \\ 0 & 0 & R_0 & 0 & 0 & R_1 & \cdots & 0 & 0 & R_{n_k}^k \end{bmatrix} \end{aligned}$$

where ${n_k}$ denotes the number of control points associated with the coupling interface of patch *k*. It should be noted that the matrices ${{\boldsymbol{K}}}_c^t$ and ${{\boldsymbol{K}}}_c^r$ correspond to the translational and rotational DOFs, respectively. Finally, the global stiffness matrix of the problem is obtained by assembling the bulk stiffness matrix ${{\boldsymbol{K}}}_b$ together with the rotational and translational coupling stiffness matrices ${{\boldsymbol{K}}}_c^r, {{\boldsymbol{K}}}_c^t$.

## Results and discussion

This section investigates the accuracy, convergence and robustness of the developed method for wing structures. First, we evaluate the convergence of isogeometric Reissner-Mindlin shell formulations using a benchmark problem. We then analyze the convergence behavior of the RAE-2822 wing using both *h*- and *p*-refinement strategies, and investigate the effect of the penalty parameter on the simulation results. Finally, the method is validated on six additional wing structures to demonstrate its generalization and robustness.

### Validation and convergence study

The isogeometric Reissner-Mindlin shell formulations are first validated using a square plate benchmark, a standard problem for evaluating plate theories in the literature^44^. The plate is defined over the domain $[0,1] \otimes [0,1]$ with a side length $L = 1$ m and is subjected to a surface load *f*(*x*, *y*). The material properties consist of a Young’s modulus $E=10.92$ MPa and a Poisson’s ratio $\nu =0.33$. The load *f*(*x*, *y*) is defined as

26 $$\begin{aligned} \begin{aligned} f(x,y) =&\dfrac{E}{12(1-\nu ^2)}[12y(y-1)(5x^2-5x+1)(2y^2(y-1)^2+x(x-1)(5y^2-5y+1)) \\&+ 12x(x-1)(5y^2-5y+1)(2x^2(x-1)^2+ y(y-1)(5x^2-5x+1))]. \end{aligned} \end{aligned}$$

The analytical solution for the transverse displacement in the *z*-direction is expressed as

27 $$\begin{aligned} \begin{aligned} U_z(x,y) =&\dfrac{1}{3}x^3(x-1)^3y^3(y-1)^3 - \dfrac{2t^2}{5(1-\nu )}[y^3(y-1)^3x(x-1)(5x^2-5x+1) \\&+x^3(x-1)^3y(y-1)(5y^2-5y+1)], \end{aligned} \end{aligned}$$

**Fig. 11:**
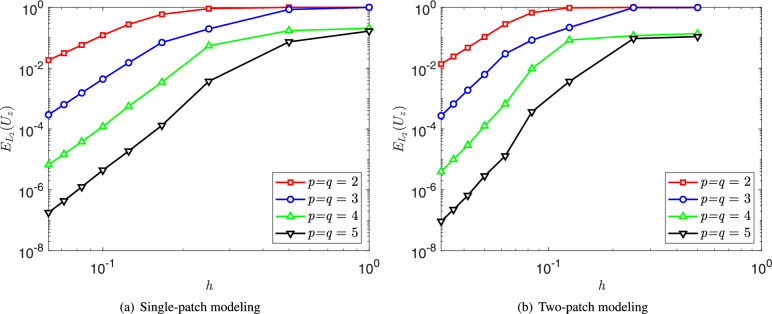
Relative $L_2$ error convergence of the vertical displacement for the square plate modeled with (**a**) a single NURBS patch and (**b**) two NURBS patches.

**Fig. 12:**
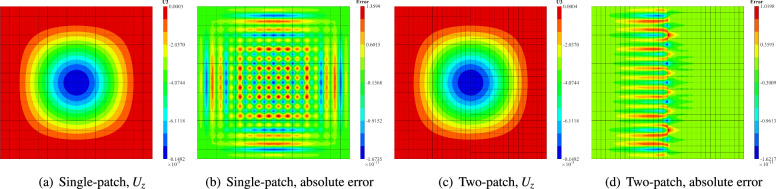
Vertical displacement contours and corresponding absolute errors for the single-patch and two-patch configurations at the finest refinement level.

To evaluate the convergence of the proposed shell formulations and the penalty-based coupling, the square plate is modeled using both single-patch and two-patch NURBS configurations. The single-patch mesh is refined sequentially through a series of grids, ranging from $1\times 1$ to $16\times 16$ elements. For the two-patch model, the left patch follows the same refinement sequence as the single-patch case, while the right patch is discretized with non-matching grids (from $1\times 2$ to $16\times 24$ elements) to create a non-conforming interface. These patches are then joined using the penalty method. Furthermore, four polynomial orders are considered with $p = q \in \{2, 3, 4, 5\}$. Convergence is assessed using the relative $L_2$ error of the vertical displacement, defined as

28 $$\begin{aligned} E_{L_2}(U_z) = \dfrac{||U_z^{ex}-U_z^h||_{\Omega }^{L_2}}{||U_z^{ex}||_{\Omega }^{L_2}} = \sqrt{ \dfrac{ \int _{\Omega }(U_z^{ex}-U_z^h)^2 d \Omega }{ \int _{\Omega } (U_z^{ex})^2 d \Omega } }, \end{aligned}$$

where $U_z^{ex}$ and $U_z^{h}$ denote the exact and numerical solutions, respectively. Figure 11 illustrates the convergence behavior for both cases, showing that the isogeometric Reissner–Mindlin shell formulations and penalty-coupling strategy achieve optimal convergence rates. Finally, the vertical displacement contours and their corresponding absolute errors at the finest refinement level are depicted in Fig. 12. The displacement contours for the single-patch and two-patch configurations exhibit excellent agreement. Furthermore, the absolute error distribution for the two-patch model illustrates that mesh density significantly influences numerical accuracy.

The constructed RAE wing structure is then utilized for isogeometric analysis. The material is assumed to be 7075 aluminium alloy, characterized by a Young’s modulus $E=71.7$ GPa, Poisson’s ratio $\nu =0.33$ and density $\rho = 2.81$ g/cm$^3$. To focus more on the geometric parametrization and analysis-suitable characteristics, the boundary conditions and load conditions are simplified. Here the wing root section is fully fixed, while the top surface of the skin is subjected to a uniform vertical traction of $T = 1 N/m^2$. The thickness of all structural components is uniformly set to 0.01 m.

**Fig. 13:**
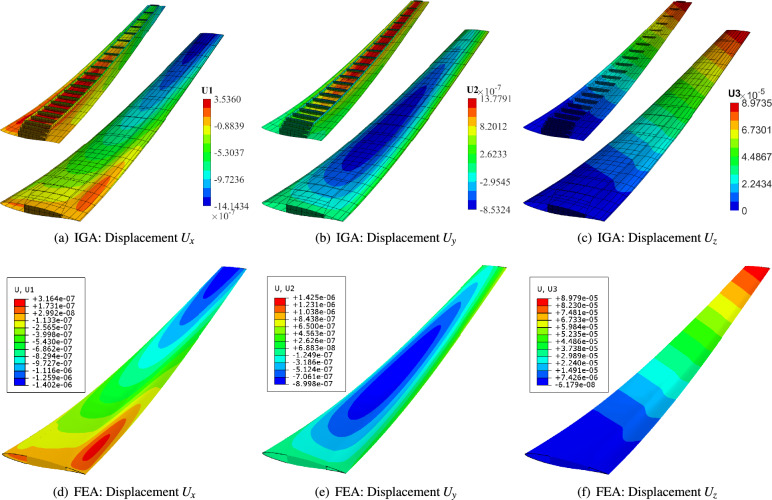
Comparison of displacement results obtained by using IGA (the upper layer) and FEA (the bottom layer). IGA uses totally 1151 biquintic NURBS elements and 9785 control points, while FEA uses 50,944 quadratic quadrilateral elements and 150,229 nodes.

For enhanced structural accuracy, the initial NURBS patches of the wing structure are refined into a total of 1151 bi-quintic elements with 9785 control points. Figure 13 compares displacement results along the three coordinate directions obtained via IGA using our in-house code and FEA performed in ABAQUS. A highly refined, conforming mesh is generated for the FEA model, consisting of 50,944 S8R elements and 150,229 nodes, approximately 15 times the number of IGA control points. The results demonstrate excellent agreement between IGA and FEA in terms of both displacement distribution and peak values. Inner views of the IGA displacement fields are also included in Figs. 12a–c , where smooth displacement contours are observed throughout.

**Fig. 14:**
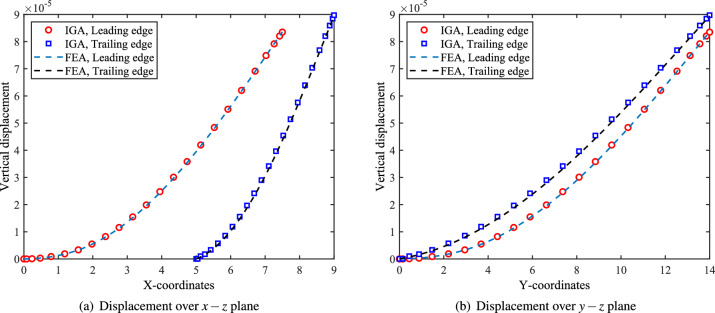
Quantitative comparison of the vertical displacements at the leading and trailing edges of the RAE2822 wing obtained using FEA and IGA.

**Fig. 15:**
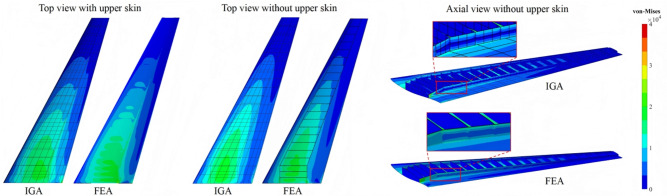
Comparison of von-Mises stress results obtained using IGA and FEA.

To quantitatively assess deformation between IGA and FEA, vertical displacements along the leading and trailing edges are plotted in the $x-z$ and $y-z$ planes, as shown in Fig. 14a,b. The displacement profiles from both methods exhibit excellent agreement. Beyond displacement, stress evaluation is essential in static structural analysis. Figure 15 presents the von Mises stress distribution on the bottom surface ($\zeta = -1$) of the shell structure from two perspectives. The left subfigures show top views with and without the upper skin, revealing strong correlation between IGA and FEA results. Notably, the IGA-derived stress field displays superior smoothness, attributable to the higher-order continuity of its basis functions. The right subfigures compare axial cross-sections. While stress values align consistently across nonconforming interfaces, reduced contour smoothness is observed, due to the $C^0$ continuity of element shape functions at patch boundaries. Overall, IGA delivers more continuous, physically consistent stress distributions.

**Fig. 16:**
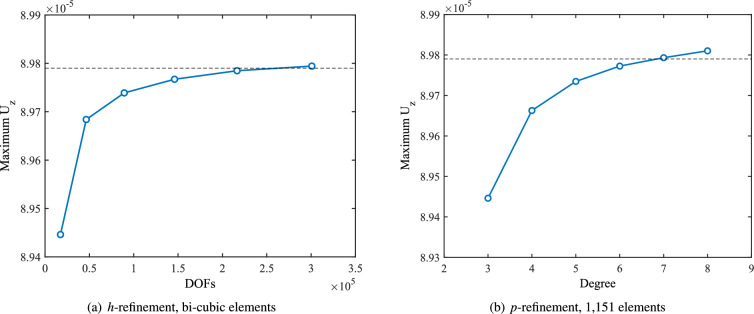
Convergence behavior of vertical displacements obtained by IGA under *h*- and *p*-refinements.

We also study the convergence of the wing’s vertical displacement $U_z$ under both *h*- and *p*-refinements strategies, as demonstrated in Fig. 16. In this figure, the dotted line represents the reference solution from a conventional FEA model using 50,944 S8R elements and 901,374 DOFs. For *h*-refinement, we start from an initial NURBS mesh of 1,151 bi-cubic elements. This mesh is then refined through five successive knot insertions, producing meshes with 4604, 10,359, 18,416, 28,775, and 41,436 elements, respectively. By the fourth refinement level (28,775 elements, 216,486 DOFs), the IGA solution has essentially reached the same accuracy as the reference FEA result. For *p*-refinement, the mesh connectivity remains fixed (1151 elements) while the polynomial degree of the basis functions is increased. Using a degree elevation algorithm, we raise the degree from bi-cubic to bi-quartic, bi-quintic, bi-sextic, bi-septic, and finally bi-octic. We find that the solution with 1151 bi-septic elements matches the accuracy of the FEA benchmark. Throughout both approaches, the wing geometry is preserved exactly, so improvements in accuracy are solely due to the refinement. Overall, both *h*- and *p*-refinement exhibit excellent convergence behavior toward the reference solution.

For a quantitative comparison, Table 1 reports the maximum displacements in the *x*, *y*, and *z* directions obtained from IGA and FEA for each case. It is noted that the maximum displacements are evaluated based on the displacements of the control points. The table also lists the number of elements and total DOFs used. These results confirm that, by using sufficiently refined IGA meshes (either through *h*- or *p*-refinement), the displacement predictions agree closely with those from the much larger FEA model.

**Table 1:** A comparison of maximum displacements in three directions obtained from IGA and FEA under different element orders.

Method	Order	#Elements	#DOFs	$-u_x^{max}$	$u_y^{max}$	$u_z^{max}$
IGA	Cubic	1151	17,358	1.423E−7	1.420E−6	8.945E−5
IGA	Quartic	1151	35,856	1.415E−7	1.398E−6	8.967E−5
IGA	Quintic	1151	58,710	1.415E−7	1.392E−6	8.974E−5
IGA	Sextic	1151	85,920	1.415E−7	1.387E−6	8.977E−5
IGA	Septic	1151	117,486	1.415E−7	1.387E−6	8.979E−5
IGA	Octic	1151	153,408	1.415E−7	1.375E−6	8.981E−5
FEA	Linear	50,944	297,918	1.384E−7	1.402E−6	8.816E−5
FEA	Quadratic	50,944	901,374	1.402E−7	1.425E−6	8.979E−5

### Effect on penalty parameter

The penalty parameter is a critical factor in penalty-based coupling of patches within isogeometric analysis. An excessively large value can induce ill-conditioning in the stiffness matrix, while an insufficiently small value may fail to adequately couple adjacent patches. In prior simulations of the RAE-2822 wing, the penalty parameters $\alpha _t$ and $\alpha _r$, defined in Eq. (24), were assigned identical values of $1 \times 10^8$. To systematically investigate its influence on displacement, we now explore twelve logarithmically spaced values ranging from $1 \times 10^1$ to $1 \times 10^{12}$ , each increased by an order of magnitude relative to the previous value.

**Fig. 17:**
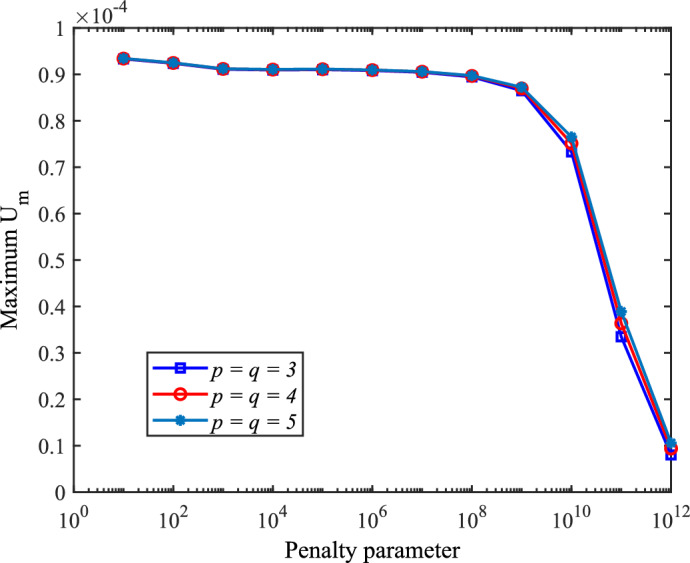
Effect of penalty parameter $\alpha$ on maximum wing displacement magnitude.

**Fig. 18:**
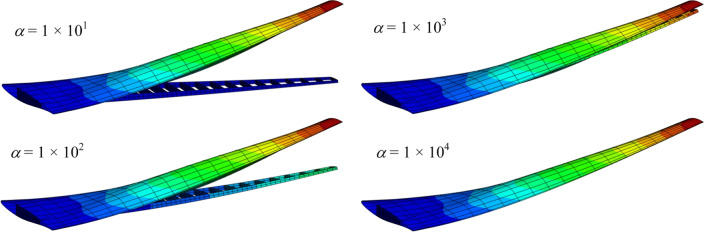
Displacement magnitude contours with penalty parameters $\alpha = 10^{1}, 10^{2}, 10^{3}, 10^{4}$. Displacements scaled by $5\times 10^4$.

Figure 17 shows the variation of maximum displacement magnitude ($U_m$) with respect to the penalty parameter $\alpha$ (where $\alpha = \alpha _t = \alpha _r$). The maximum $U_m$ remains stable for $\alpha \le 10^9$ but decreases sharply for $\alpha > 1 \times 10^9$. Although small $\alpha$ values ($1 \times 10^1, 1 \times 10^2, 1 \times 10^3$) yield visually similar maximum displacements, they fail to couple the patches, as evidenced by the displacement contours in Fig. 18. These contours clearly show separation between the wingbox and skin. As $\alpha$ increases, this discrepancy progressively diminishes. Note that displacements are magnified by a scale factor of $5 \times 10^4$ to enhance visualization of the separation phenomenon.

### Extension to other wing structures

**Fig. 19:**
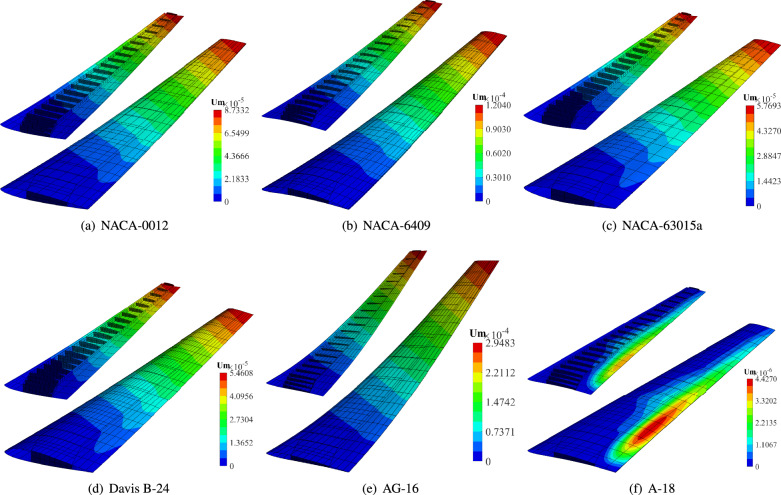
Displacement magnitude of the six wing structures obtained through isogeometric analysis. The top skin surface is hidden to reveal internal details. Displacements are scaled by a factor $2\times 10^4$.

In addition to the RAE2822 wing, we performed isogeometric analysis on six wing structures based on the NACA-0012, NACA-6409, NACA-63015a, Davis B-24, AG-16, and A-18 airfoils (Fig. 10). Identical material properties, boundary conditions, and loading conditions were applied to those used for the RAE-2822 wing. Figure 19 shows the resulting displacement magnitude contours obtained using our isogeometric framework. To visualize internal displacement distributions, the top skin surfaces are omitted. Displacements in all directions are uniformly scaled by a factor of $2\times 10^4$. The AG-16 wing exhibited the largest deformation, with a maximum displacement magnitude of $2.9483\times 10^{-4}$ m. Conversely, the A-18 wing proved significantly stiffer, showing a maximum displacement of only $4.4270\times 10^{-6}$ m. However, local buckling was observed near the wing root and trailing edge on its top and bottom skins. The displacement patterns for the NACA-0012, NACA-63015a, and Davis B-24 wings were similar to that of the RAE-2822 wing. To facilitate comparison, Table 2 presents the maximum displacement magnitudes for the wing structures corresponding to the six airfoils.

**Table 2:** Maximum displacement magnitudes of wing structures corresponding to the NACA-0012, NACA-6409, NACA-63015a, Davis B-24, AG-16, and A-18 airfoils.

Displacement	NACA-0012	NACA-6409	NACA-63015a	Davis B-24	AG-16	A-18
$U_m$	8.7332E−5	1.2040E−4	5.7693E−5	5.4608E−5	2.9483E−4	4.427E−6

While the present study is strictly confined to static bending analysis of wing structures within a solid mechanics framework, it should be noted that recent advances have demonstrated isogeometric analysis’s superior accuracy in computational aerodynamics^46,47^. This naturally motivates future research directions toward integrating isogeometric analysis with computational fluid dynamics for high-fidelity aerodynamic shape optimization. It should be noted that while penalty-based coupling is effective for static analysis in this paper, it may introduce spurious eigenfrequencies in a dynamic context. A detailed investigation into the stabilization or mass-scaling techniques required to mitigate these numerical artifacts remains a subject for future research. In addition, wing structures may undergo large deformations with small strains during actual flight. Therefore, accounting for geometric nonlinearity^48–50^ warrants further investigation.

## Conclusion

A NURBS based shape parametrization framework is developed to construct analysis-suitable wing structures and to evaluate their mechanical performance using isogeometric analysis. The Reissner–Mindlin shell theory, which can readily handle the $C^0$ continuity of the geometry, is employed for multi-patch structural analysis. A combined conforming/nonconforming approach is employed to perform isogeometric analysis of complex wing structures. Non-matching interfaces between the skin and wingbox are coupled using a penalty method to enforce displacement continuity. Comparison of the numerical results with those obtained using ABAQUS confirms the accuracy and effectiveness of the developed method. Due to the high-order, high-continuity nature of the shape functions, isogeometric analysis can achieve comparable accuracy with significantly fewer DOFs, thereby improving computational efficiency. The proposed framework has been successfully implemented in constructing aircraft wing structures with seven distinct airfoil profiles and can be readily extended to accommodate additional airfoil geometries and wing planforms.

## Data Availability

The data are available from the corresponding author upon request.
